# Invisible Warriors in the Struggle Against Cancer: Social Support and Spiritual Care—A Phenomenological Study on Patient Experiences †

**DOI:** 10.3390/healthcare13162023

**Published:** 2025-08-16

**Authors:** Esma Özmaya, Sevda Uzun

**Affiliations:** 1 Department of Child Development, Faculty of Health Sciences, University of Karamanoğlu Mehmetbey, 70100 Karaman, Turkey; 2 Department of Psychiatry Nursing, Faculty of Health Sciences, Gümüşhane University, 29100 Gümüşhane, Turkey; sevdauzun50@gmail.com

**Keywords:** cancer, social support, spiritual care, phenomenological study

## Abstract

**Objective:** The aim of this study is to examine the effect of social support on the mental state of cancer patients using a phenomenological approach. **Materials–Methods:** In this study, a phenomenological research orientation was used, and semi-structured in-depth interviews were conducted with 14 people diagnosed with cancer living in a province in central Turkey. The criterion sampling method, one of the purposeful sampling methods, was used for the sample group. The interviews continued until data saturation was achieved, and all interviews were recorded and later transcribed verbatim. The data were analyzed using thematic analysis, and the study was conducted and reported according to the COREQ checklist. **Results:** Two categories (effects of cancer and needs of the cancer patient (invisible components)) and five themes (psychological effects, social effects, physical effects, social support, and spiritual care) were identified in the analysis of the data. **Conclusions:** It has been determined that individuals are affected by cancer mentally, physically, and socially, and have difficulty coping. In particular, it has been found that social support and thinking about the purpose of life, supporting hope, and self-acceptance are quite important in increasing individuals’ spirituality.

## 1. Introduction

According to the latest estimates on the global cancer burden by the World Health Organisation’s (WHO) cancer agency, the International Agency for Research on Cancer (IARC), 9.7 million cancer-related deaths occurred in 2022, and one in five people is battling cancer [1]. Individuals with cancer experience various symptoms (physical symptoms such as fatigue, loss of appetite, shortness of breath, coughing, pain, and bloody sputum, as well as psychological distress such as irritability, sleep disturbances, sadness, hopelessness, depression, and anxiety) caused by both the disease and its treatment [2,3]. The processes associated with the diagnosis and treatment of cancer, profoundly impacting the lives of patients and their families, can induce significant psychological stress. This stress arises from alterations in personal lives, daily activities, work, relationships, and family roles. Throughout the psycho-social journey related to cancer, the anxieties experienced by patients have the potential to strain social relationships, necessitating support from their environment [4,5].

Social support, a pivotal factor influencing individuals’ experiences with their illnesses, refers to the assistance received from those around them when confronted with stressful situations. While social support implies the presence of individuals capable of offering support or aid in the patient’s social environment, it also reflects the quality and quantity of social relations. Social support comprises four components: emotional, instrumental, informational, and appraisal support. Particularly in diseases like cancer, emotional, instrumental, and informational support hold significant importance [5,6,7]. Emotional support aids individuals in coping with the emotional challenges of their situation, thereby enhancing their resilience. Instrumental support provides practical and concrete assistance with daily tasks. Informational support helps individuals navigate the challenges related to the disease and treatment process, fostering a sense of control through advice obtained from others who have faced similar situations. Studies indicate that social support plays a crucial role in crises, influencing the mental and physical health outcomes of individuals. Particularly in diseases such as cancer, social support is deemed a vital resource for facilitating psychological adaptation. The literature further emphasizes the positive effects of social support on the overall well-being and quality of life of cancer patients [8,9].

Individuals suffering from life-threatening diseases, particularly cancer, require spiritual care. Beyond causing physical discomfort, cancer also affects individuals’ mental well-being and leads to deep and difficult problems. Cancer, being a persistent and potentially fatal illness with unpredictable symptoms, progressive deterioration, and lasting effects, prompts individuals to reassess their lives from a new perspective [10,11].

Upon receiving a cancer diagnosis, individuals often grapple with questions such as “Why me?” “Is the Creator punishing me?” and seek spiritual support by questioning the meaning of life [11]. A study on spiritual needs among cancer patients in Türkiye revealed that individuals sought answers to questions about the moment of death and the afterlife (100%), desired to feel peaceful and content (94.8%), sought companionship throughout the process (93.5%), wished to have others pray for them (52.2%), and sought compassion and kindness (54.3%) [12]. The relevant literature highlights the importance of the perception of social support and spiritual care among cancer patients [10,11,12]. In this context, this study aims to phenomenologically evaluate the life experiences of cancer patients, focusing on their perceptions of social support and spiritual care needs.

## 2. Methodology

In this study, the authors adhered to the Consolidated Guidelines for Reporting Qualitative Research (COREQ) (https://www.equator-network.org/aboutus/equator-network-what-we-do-and-how-we-are-organised/, accessed on 7 August 2025) and ensured that the research processes were reported comprehensively (Table 1) [13].

## 3. Study Design

This study was conducted between July 2023 and September 2023, employing an inductive qualitative design. Semi-structured, in-depth individual interviews were conducted with 14 individuals diagnosed with cancer, living in a province in the central region of Türkiye, and undergoing chemotherapy at a state hospital.

### 3.1. Research Team and Reflexivity

Both members of the research team are active faculty members with expertise in psychiatric nursing, holding doctoral degrees. They have clinical nursing experience and have received training in qualitative research methods. Both researchers have conducted different qualitative studies with cancer patients.

### 3.2. Recruitment and Participants

In the study, criterion sampling was used as a purposeful sampling method for the sample group. Criterion sampling involves selecting individuals, events, or situations with specific characteristics related to the research problem [14,15].

The inclusion criteria consist of individuals (a) diagnosed with cancer, (b) undergoing chemotherapy, (c) being able to communicate and wanting to communicate (d) willing to participate. Exclusion criteria included individuals (a) with language, speech, or hearing impairments hindering communication and (b) unwilling to participate.

### 3.3. Validity and Reliability of the Research

Four basic criteria were taken into consideration to ensure the reliability and validity of the data obtained in this research. In order to increase the credibility of the research, the findings obtained from the interview analyses were shared with two participants with a high level of education, and their approval was obtained. Thus, the accuracy of the data was tested from the participants’ perspective [16].

In order to demonstrate the applicability of the findings in other contexts and to meet the transferability criterion, detailed descriptions of the participant profiles and the environment in which the research was conducted were provided. This suggests that similar results could be achieved in other studies conducted under similar conditions.

Each step of the research process was explained in detail to ensure reliability. In addition, in accordance with the principle of verifiability, the participants’ statements were directly included before the analysis. This ensured that the data on which the research findings were based were presented transparently.

To support the internal reliability of the data, the coding process was carried out independently by two different researchers. To increase internal validity, the questions in the semi-structured interview form were shaped based on feedback from four experts in the field, and the findings were evaluated in line with the opinions of another expert.

### 3.4. Data Collection

Based on a review of the literature, the researchers designed a semi-structured interview form. This interview form was prepared by two authors who are experts in the field (psychiatric nursing) and finalized after obtaining expert opinions from two researchers who are experts in the field of internal medicine.

This form consists of two sections, the first of which contains questions about age, gender, marital status, occupation, socioeconomic status, and the length of time the person has been a cancer patient. The second section features eight fundamental open-ended questions designed for use in the semi-structured interviews. These interview questions were used in face-to-face interviews with individuals [17]. During these interviews, the participants were encouraged to articulate their perspectives on the impact of cancer, their views on social support, and their spiritual needs. Additionally, prompts such as “Could you elaborate further?” and “What do you mean?” were utilized. The interviews were exclusively conducted by the second author, recorded via a Sony voice recorder, and meticulously transcribed by the same two researchers. After the completion of all interviews, the data underwent transcription for subsequent analysis.

Questions in the semi-structured interview form were as follows:

1. How did you feel when you were diagnosed with cancer?
  (a) Which emotions did you experience?
  (b) What did you think?
  (c) What are your experiences?
2. What does the word cancer remind you of? What does it mean to you? (After the participant answers, “Is it curable? What are the effects of cancer on individuals?”).
3. How did you feel when you were diagnosed with cancer? Which emotions did you experience? What did you think? What are your experiences?
4. How did you initially disclose your situation, and with whom did you choose to share it first? What reactions did your spouse and/or relatives have, and how did you respond to these reactions?
5. Regarding your cancer diagnosis, do you openly communicate about it? What are the reasons?
6. How does society view cancer patients? What do you think about this issue?
7. How did you manage the process? What are the situations that contributed positively or negatively to your successful or unsuccessful management of the process? What do you think about your sources of support in this process? Do you think you are in the process of adaptation? What are the reasons?
8. What are the needs of cancer patients in the community? What would you like to have as social support?
9. Why is spirituality important? What would you like to have in this regard? What gives you spiritual strength? What do you expect from nurses and society in this regard?

## 4. Data Analysis

Qualitative data analysis of the interview findings employed Colaizzi’s seven-stage analysis method for phenomenological studies [18]. Two researchers independently read the interview transcripts to understand the information conveyed. Then, the data underlying these statements was identified and analyzed. The researchers discussed and verified the meanings until a consensus was reached. The themes were then identified and organized into main themes and sub-themes. Through extensive discussions resulting in a consensus, the researchers formulated and validated the meanings derived from the data. Subsequently, they categorized these insights into main themes and sub-themes. These themes and sub-themes were crafted via a lucid narrative structure. Furthermore, participant statements were integrated to enable readers to authenticate the interpretation and analysis of the data [19,20,21].

### Ethical Considerations

Approval for this research was obtained from the X University Scientific Research and Publication Ethics Committee (dated 29 March 2023, numbered E-11095095-050.01.04-122725). Informed consent was received from the participants before interviews, and recordings and transcripts were stored on a password-protected device. The study adhered to the principles of the Declaration of Helsinki and the ethical standards of the National Research Committee.

## 5. Results

The sociodemographic characteristics of the participants are presented in Table 2.

As a result of the analysis of the data from semi-structured interviews, categories, themes, and sub-themes were determined (Table 3).

**Category 1. Effects of cancer**

**Theme 1. Psychological effects**

Based on interview data, individuals expressed a range of emotional responses to cancer, including confusion, loneliness, anxiety, fear of death, learning patience, weakness, future anxiety, sadness or unhappiness, helplessness, and burnout. The participants shared their perspectives:

*“Cancer evokes death. It is difficult to cope. There is a treatment for some cancers; for example, I know that there are people like me who have breast cancer and recovered, but at first, you feel uneasy when you hear it.”*

(P2)

*“The word cancer evokes words like pain, loneliness, and death. Some types of cancer can be cured, and early diagnosis is also important. This disease causes great devastation psychologically and physically in every aspect; fear, anxiety, life order changes, sometimes you can’t even dream about the future; this is a very bad thing, such as nutritional problems or sleep problems.”*

(P8)

**Theme 2. Social effects**

The participants reported various social challenges, such as the need for family support, fear of being alone, learning to cope with side effects, and struggling to prevent infection. Their experiences were reported as follows:

*“When I first learned about it, I took it normally. I made my best effort without demoralizing myself. I tried not to be defeated. I researched doctors and what to eat and drink; I tried to keep my morale high; I tried not to neglect my work and my family. But recently, I’ve started to feel tired. The fact that there was no progress and it had metastasized affected me very badly, both psychologically and socially.”*

(P10)

*“I first told my husband about the cancer diagnosis, and then my parents found out, of course. Actually, they all gave me hope and supported me; I can’t blame them, but after a while, I started to feel that they were tired too. They don’t have complaints about me; that’s how I feel, or maybe I don’t want to be a burden on them anymore.”*

(P6)

**Theme 3. Physical effects**

Interviews revealed that the participants faced physical challenges such as loss of appetite, insomnia, fatigue, and weakness due to cancer.

*“I had nausea, vomiting and hair loss; it very bad…”*

(P3)

*“People normally hide this bad disease from their spouses, friends, and relatives, but I suddenly told my husband. I think it was better that he learnt it. After all, he was going to find out somehow, so I thought he should know and be with me. Later, it was already very bad: fatigue, weakness, I had sores in my mouth, I lost my hair… My husband was going to find out anyway…”*

(P6)

**Category 2. Needs of the cancer patient (invisible components)**

**Theme 1. Social support**

The participants expressed the significance of various forms of social support, including support from family and friends, affection from pets, assistance from neighbors, and a belief in divine intervention. One participant emphasized the importance of social support, as follows:

*“I don’t want to sound demanding, but it’s better to get support instead of people feeling sorry for you. Dealing with this stuff messes with your daily life and mood–one moment you’re mad, and the next you might be happy. My friends wished me well, which was nice, but I wished they’d do more, like send flowers, call to check in or invite me for coffee. My family was always there, but some extra support from friends would’ve been great.”*

(P6)

*“In this illness, sincerity is important. In other words, support should be given to us because our life continues as it is. We shouldn’t be looked at as if we’re going to die. Our daily lives are ongoing, and they have to go on somehow. Constantly thinking about the illness has a very negative psychological impact. So, chatting with friends, carrying on with your work, doing what you can—these are necessary.”*

(P2)

**Theme 2. Spiritual care**

In expressing their spiritual care requirements, the participants emphasized the importance of healthcare professionals responding to their inquiries and offering companionship rather than leaving them alone. They sought answers regarding death, desired kindness, and expressed a need to engage in religious worship while in the hospital. Additionally, the participants valued friendly interactions, wished to experience the love of their pets, and anticipated understanding and empathetic behavior from both healthcare personnel and family members. They also highlighted the significance of seeking prayer support from others, finding joy in remembering the past, dreaming about the future, engaging in prayer, attributing the illness to a higher power, and ultimately accepting it.

*“Spirituality means inner peace. I think it’s important to achieve inner peace, but I haven’t found that inner peace yet. For me, some memories from my childhood help with this—thinking about the good memories and trying to feel the emotions of those moments. Daydreaming also helps. Right now, I’m a lonely person, but remembering my childhood spent in a large family environment feels good. I’m not a strongly religious person, so I won’t say much about that aspect.”*

(P14)

*“Spirituality, for me, is being with my loved ones. Having my loved ones around gives me strength. My children, and grandchildren—I mentioned earlier that loneliness is tough, especially with this illness. So, when they’re with me, I often forget about the illness. Doctors and nurses should be more attentive and listen to patients who experience loneliness. Just asking about their well-being from time to time is enough.”*

(P1)

## 6. Discussion

The study focused on evaluating cancer patients’ perceptions of their life experiences and their needs for social support and spiritual care. Individuals’ life experiences were divided into two categories.

### 6.1. Effects of Cancer

Despite advancements in health-related treatments and technology for diagnosing and treating cancer, the prolonged nature of the treatment process, the financial burden it imposes, the loss of body image, anxiety related to the potential spread of the disease to distant organs, and the realization of numerous surgical procedures present significant challenges for patients and their families. Cancer is a disease that affects individuals not only physically but also mentally. The medications and other treatments used cause fatigue and various difficulties in economic, social, emotional, and physical terms. The struggles stemming from these multifaceted challenges profoundly impact both the individual undergoing treatment and their family. The transformative impact of cancer extends beyond the diagnosed patients, affecting their families and close relatives [22,23].

The perception of cancer in society, widespread fear and anxiety about the future, and stress related to experiences that may be encountered during the course of the disease and treatment have a more negative impact on patients and their loved ones than other disease groups [24]. Beyond being solely a physical ailment, cancer transcends a singular life event with a definitive end linked solely to medical treatment. Instead, this diagnosis not only leaves a lasting effect on the psycho-social aspect of the individual due to the profound uncertainty experienced during and after the treatment process but also induces a multidimensional life experience, leading to meaningful changes [24,25]. The results of the study are consistent with the existing literature and emphasize that patients battling cancer are negatively affected psychosocially.

### 6.2. Needs of the Cancer Patient (Invisible Components)

Social support, including support from family, friends, and institutions to bolster an individual’s psychological well-being and offer both financial and emotional aid, plays a crucial role in shaping human behavior. Perceived social support not only protects and reinforces one’s sense of identity but also fulfills the need for connection. Maintaining the psychological and social well-being of individuals, navigating challenges, fostering positive health behaviors, strengthening social support networks, and determining self-efficacy are integral aspects of the support received for counseling purposes [26].

In a study by İsmailoğlu et al. (2016) involving patients with head and neck cancer, it was found that the ability to cope with stress positively correlated with increased mean scores of social support, leading to an enhancement in the quality of life [27]. Particularly for patients diagnosed with breast cancer, social support from spouses and family emerges as a crucial factor in coping with the disease and adapting psychosocially [28]. A study conducted by Rong et al. (2022) found that social support is associated with spirituality and well-being [5]. Our research supports these findings and shows the importance of social support for cancer patients.

Religion and spirituality serve as vital resources for cancer patients, offering coping mechanisms during diagnosis and treatment. In a comprehensive study of patients with chronic illnesses, a positive relationship between spirituality and the ability to cope with chronic conditions was identified. Spirituality and belief in a higher power contribute to making challenging circumstances more bearable [29,30].

The role of spirituality is emphasized in individuals’ coping with diseases, ensuring well-being, and influencing the treatment and recovery process of chronic diseases. Atan et al. (2020) [31] indicated that spirituality has a positive impact on mental health in cancer patients, with high spiritual beliefs correlating with higher levels of hope. Patients with a strong spiritual connection tend to exhibit better psychological well-being, increased optimism, lower stress and anxiety levels, greater hopefulness, and a reduced likelihood of attempting suicide compared to those with lower spirituality levels [31]. Patients undergoing cancer treatment care about spiritual care, often expecting support, especially from healthcare professionals. The study suggests that individuals negatively affected by cancer, with established social support and a reliance on spiritual coping strategies, tend to adapt more effectively to the challenges posed by the disease.

**Limitations:** The study has certain limitations. All the participants were drawn from a specific province in the central region of Türkiye; therefore, the findings are limited to the characteristics of the participants and the context in which the study took place and may not fully represent the diverse population of individuals dealing with cancer.

## 7. Conclusions

Cancer emerges as a comprehensive health challenge, impacting various facets of individuals’ lives. The study revealed that individuals experienced mental, physical, and social outcomes due to cancer, encountering difficulties in coping. The manifestation of symptoms had a significantly adverse effect on individuals, prompting diverse coping methods that, at times, proved insufficient. The challenges encountered had a detrimental impact on the overall quality of life.

Despite all the challenges, the patients demonstrated a positive approach to managing the coping process, notably through the utilization of spiritual coping strategies. Social support, contemplation of life’s purpose, fostering hope, and self-acceptance were identified as crucial elements in enhancing individuals’ spirituality. Recognizing the significance of these aspects, it is believed that assessing and addressing the spiritual needs of cancer patients plays a pivotal role in promoting their overall well-being.

## Figures and Tables

**Table 1:** Combined criteria for reporting qualitative research (COREQ).

**Domain 1: Research team and reflexivity.**			
**Personal Characteristics**			
**Number**	**Characteristics**	**Guiding Questions**	**Explanations**
1	Interviewer/facilitator	Which author(s) conducted the interview or focus group?	The first author conducted the interview.
2	Credentials	What were the credentials of the researchers? (e.g., Ph.D., MD)	First author: Ph.D. Second author: Ph.D.
3	Occupation	What was their occupation during the study?	First author: Dr. Faculty Member, Psychiatric Nursing Second author: Dr. Lecturer, Psychiatric Nursing
4	Gender	What was the sex of the researcher?	Two researchers Female
5	Experience and education	What are the experiences and education levels of the researchers?	The first author has taken qualitative courses, has experience in qualitative research, and has published qualitative studies in international journals. The second author has taken qualitative courses, has experience in qualitative research, and has published qualitative studies in international journals.
**Relationships with Participants**			
6	Relationship status	Was there a relationship between the researcher and the participants before the training?	No relationship was established before the study.
7	Interviewee’s information about the interviewer	What did the participants know about the researcher, e.g., personal goals and reasons for doing the research?	Participants knew that the researcher had a doctorate in the field of mental health and diseases.
8	Interviewee characteristics	What characteristics of the interviewer or facilitator were reported? (e.g., bias, assumptions, reasons, and interests in research)	At the beginning of each interview, participants were informed about the purpose and objectives of the study.
**Domain 2: Study Design**			
**Theoretical Framework**			
9	Methodological orientation and theory	What methodological orientation was identified to support the study, e.g., discourse analysis, ethnography, phenomenology, and content analysis?	This was a qualitative study.
**Sampling**			
10	Sampling	How were the participants selected? (e.g., purposeful, convenience, consecutive, snowball.)	The criterion sampling method, one of the purposive sampling methods, was used.
11	Approach method	How were the participants reached? (e.g., face-to-face, telephone, mail.)	The time of the interviews was scheduled by the students who voluntarily agreed to participate in the study.
12	Sample size	How many participants were there in the study?	A total of 14 individuals were included in the study.
13	Exclusion	How many people refused to participate or dropped out? Reasons?	No adolescents refused to participate in the study.
**Setting**			
14	The setting of data collection	Where were the data collected? (e.g., home, clinic, or workplace)	Detailed information is given in the data collection section of the study.
15	Presence of non-participants	Was there anyone else other than the participants and the researchers?	No, there was not.
16	Description of the sample	What are the important characteristics of the sample? (e.g., demographic data, date)	Individuals who agreed to participate in the study were included in the study.
**Data Collection**			
17	Interview guide	Were questions, prompts, and guidelines provided by the authors? Were they tested in a pilot study?	Detailed information is given in the methods section.
18	Repeat interviews	Were repeated interviews conducted? If yes, how many?	No, they were not.
19	Audio/visual recording	Was audio recording or visual recording used to collect data in the research?	The responses of all individuals and the researcher’s observations were recorded.
20	Field notes	Were field notes taken during and/or after the interview or focus group?	Each interview lasted between 35 and 45 min.
21	Duration	How long were the interviews or focus groups?	Yes, it was.
22	Data saturation	Was data saturation discussed?	No, they were not.
23	Transcripts returned	Were transcripts returned to participants for comment and/or correction?	The responses of all individuals and the researcher’s observations were recorded.
**Domain 3: Analysis and Results**			
24	Number of data coders	How many data coders coded the data?	Two researchers and a third individual coded the data.
25	Description of the coding tree	Did the authors describe the coding tree?	The titles and subtitles in the results section represent the final coding tree.
26	Derivation of themes	Were the themes predetermined or derived from the data?	Themes were derived from the data.
27	Software	If any, what software was used to manage the data?	The data were analyzed manually.
28	Participant control	Did participants provide feedback on the findings?	No, they did not.
**Reporting**			
29	Quotations provided	Are participant quotes cited to illustrate themes or findings? Is each quote identified, e.g., by participant number?	Yes, they are. Participant quotes are provided to illustrate themes or findings. (e.g., participant number)
30	Data and findings consistent	Was there consistency between the data presented and the findings?	Yes, there was.
31	Clarity of the main themes	Are the main themes presented in the findings?	Yes, they are.
32	Clarity of subthemes	Is there a description of the different cases or a discussion of minor issues?	Yes, there is.

**Table 2:** Characteristics of the participants.

Participant Number	Age	Gender	Marital Status	Education Level	Occupation	Income Level	Duration of the Illness	Type of the Illness	Place of Residence
P1	65	Female	Single	Primary school	Housewife	Income equal to expenses	2 years	Breast	City
P2	32	Male	Single	High school	Unemployed	Income less than expenses	1 year	Testis	District
P3	44	Male	Married	Primary school	Self-employed	Income less than expenses	1.5 years	Intestine	District
P4	51	Erkek	Single	Primary school	Unemployed	Income equal to expenses	2 years	Intestine	City
P5	65	Male	Single	Primary school	Self-employed	Income equal to expenses	3 years	Stomach	City
P6	56	Female	Single	High school	Housewife	Income less than expenses	2 years	Breast	City
P7	42	Male	Single	Primary school	Unemployed	Income less than expenses	2 years	Intestine	District
P8	24	Female	Single	High school	Self-employed	Income more than expenses	1 year	Lymph gland	District
P9	38	Male	Married	High school	Civil servant	Income less than expenses	3 years	Soft tissue	City
P10	42	Female	Single	High school	Self-employed	Income less than expenses	5 years	Breast	City
P11	31	Male	Single	University	Unemployed	Income more than expenses	1 year	Lymph gland	City
P12	44	Female	Married	Primary school	Unemployed	Income less than expenses	2 years	Ovary	City
P13	58	Male	Single	Primary school	Self-employed	Income less than expenses	7 years	Stomach	District
P14	32	Male	Married	Secondary school	Tradesman	Income less than expenses	1 year	Testis	District

**Table 3:** Social support perceptions and spiritual care needs of cancer patients.

Categories	Themes	Subthemes
**1. Effects of cancer**	**A. Psychological effects**	A1. Confusion
		A2. Loneliness
		A3. Anxiety
		A4. Fear of death
		A5. Learning patience
		A6. Weakness
		A7. Future anxiety
		A8. Sadness/unhappiness
		A9. Despair
		A10. Burnout
	**B. Social effects**	B1. Needing family support
		B2. Fear of being alone
		B3. Learning to cope with side effects
		B4. To fight against infection
	**C. Physical effects**	C1. Fatigue
		C2. Pain
		C3. Nausea
		C4. Fatigue
		C5. Hair loss
		C6. Weakness
		C7. Weight loss
		C8. Aphthae in the mouth
		C9. Sleep problems
		C10. Loss of appetite
		C11. Decreased quality of life
**2. Needs of the cancer patient (invisible components)**	**A. Social support**	A1. Family support
		A2. Friend support
		A3. Love of pets
		A4. Support from neighbors
		A5. Believing that God is helping him
	**B. Spiritual care**	B1. Health personnel answering the questions
		B2. Health personnel being with the individual
		B3. Not to be left alone
		B4. Getting answers to questions about death
		B5. Showing courtesy
		B6. Being able to fulfill religious worship in the hospital
		B6. Friendly health personnel
		B7. Feeling the love of a pet
		B8. Expecting understanding from health personnel and family members
		B9. Expecting empathic behavior from health personnel
		B10. Asking other people to pray for him/her
		B11. Thinking about the good old days and being happy
		B12. Dreaming about the future
		B13. Praying
		B14. Believing that the disease comes from God and accepting it

## Data Availability

Data supporting the findings of this study are available from the corresponding researcher, and raw data are available upon request.
